# The feather pattern *autosomal barring* in chicken is strongly associated with segregation at the *MC1R* locus

**DOI:** 10.1111/pcmr.12975

**Published:** 2021-05-04

**Authors:** Doreen Schwochow, Susanne Bornelöv, Tingxing Jiang, Jingyi Li, David Gourichon, Bertrand Bed’Hom, Ben J. Dorshorst, Cheng-Ming Chuong, Michèle Tixier-Boichard, Leif Andersson

**Affiliations:** 1 Department of Animal Breeding and Genetics, Swedish University of Agricultural Sciences, Uppsala, Sweden; 2 Université Paris-Saclay, INRAE, AgroParisTech, GABI, Jouy-en-Josas, France; 3 Science for Life Laboratory, Department of Medical Biochemistry and Microbiology, Uppsala University, Uppsala, Sweden; 4 Department of Pathology, University of Southern California, Los Angeles, CS, USA; 5 Department of Animal and Poultry Sciences, Virginia Tech, Blacksburg, VA, USA; 6 Department of Veterinary Integrative Biosciences, College of Veterinary Medicine and Biomedical Sciences, Texas A&M University, College Station, TX, USA; 7 INRAE, PEAT, Nouzilly, France

**Keywords:** chicken, feather patterning, genetics, MC1R, pigmentation, SOX10

## Abstract

Color patterns within individual feathers are common in birds but little is known about the genetic mechanisms causing such patterns. Here, we investigate the genetic basis for autosomal barring in chicken, a horizontal striping pattern on individual feathers. Using an informative backcross, we demonstrate that the *MC1R* locus is strongly associated with this phenotype. A deletion at *SOX10*, underlying the dark brown phenotype on its own, affects the manifestation of the barring pattern. The coding variant L133Q in *MC1R* is the most likely causal mutation for autosomal barring in this pedigree. Furthermore, a genetic screen across six different breeds showing different patterning phenotypes revealed that the most striking shared characteristics among these breeds were that they all carried the *MC1R* alleles *Birchen* or *brown*. Our data suggest that the presence of activating *MC1R* mutations enhancing pigment synthesis is an important mechanism underlying pigmentation patterns on individual feathers in chicken. We propose that MC1R and its antagonist ASIP play a critical role for determining within-feather pigmentation patterns in birds by acting as activator and inhibitor possibly in a Turing reaction–diffusion model.

## 1 | INTRODUCTION

**FIGURE 1:**
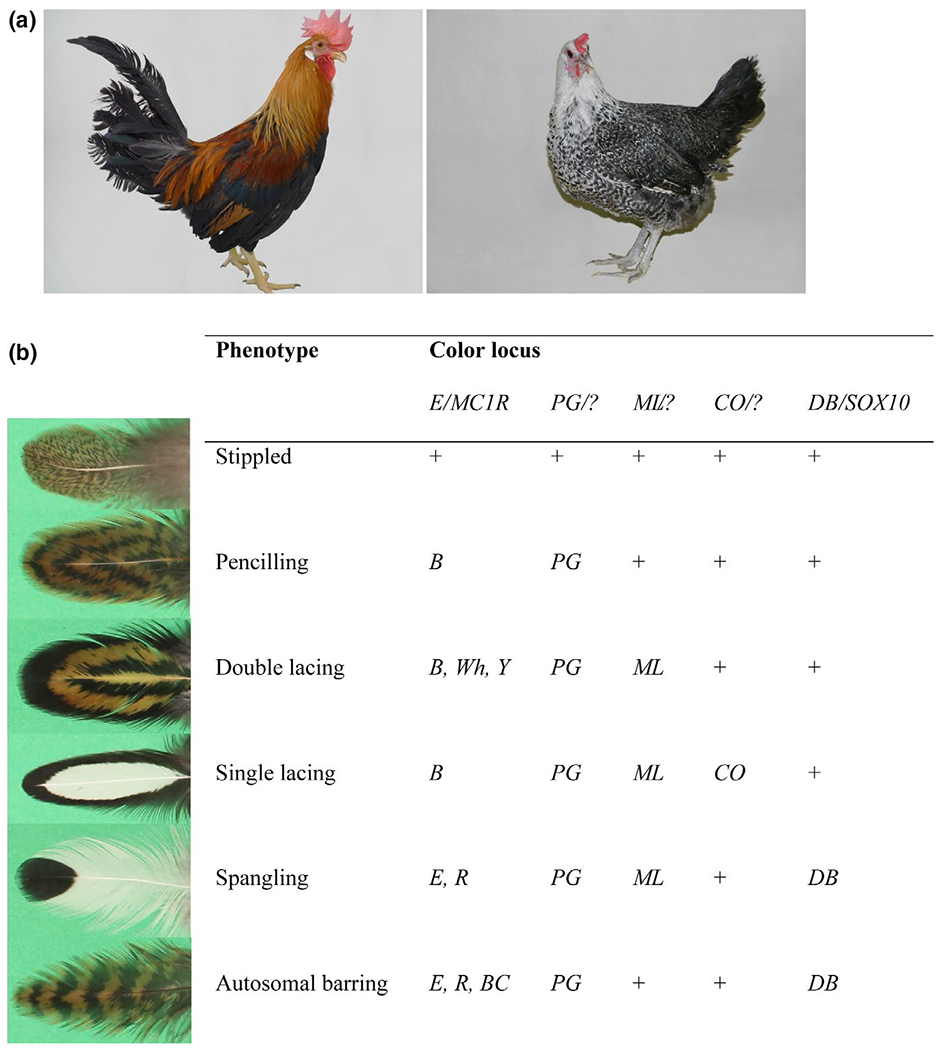
Phenotypic characterization. (a) Pigmentation phenotypes of the breeds used to generate the intercross, Light Brown Leghorn (left) showing wild-type color and Fayoumi (right) showing autosomal barring. Photograph credit: Laurence Verrier for the LBL male and David Gourichon for the Fayoumi female. (b) Within-feather pigmentation pattern in chickens. The proposed major *Patterning* (*Pg*) locus interacts with several other loci causing complex variation in melanin distribution across individual feathers. Wild-type alleles are indicated by “+.” *E*/*MC1R*–*Extension*/*Melanocortin-1 receptor, Ml*–*Melanotic*, *Co*–*Columbian*, *Db*–*Dark Brown*/*SOX10*. Modified after Andersson et al. (2020)

Pigmentation variation among birds is astonishing and is a consequence of the ability to create complex patterns through varied pigment deposition across the body as well as on individual feathers. Due to the usual homogeneous plumage appearance within species, it is challenging to pinpoint the underlying genetic cause for the observed phenotype diversity among species. The domestic chicken is a widely used model for genetic studies of the bewildering pigment variation in birds and has been used successfully so in the past (Andersson et al., 2020). Chickens exhibit a variety of within-feather pigmentation patterns of which five have been described to be the consequence of the interaction between a proposed major locus called *Patterning* (*Pg*) and other loci (Figure 1; Figure S1) (Smyth Jr, 1990). Two of these interacting loci, *Extension (E)* and *Dark brown* (*Db*), have been identified at the molecular level. *E* corresponds to the *melanocortin 1 receptor* gene (*MC1R*), one of the major pigmentation genes in vertebrates (see below). *Db* is caused by a 8.3 kb deletion 14 kb upstream of exon 1 in *SOX10* on chromosome 1 (Gunnarsson et al., 2011). *Pg* is assumed to affect the distribution of dark pigment on individual feathers and to underlie penciling, which is characterized by elliptic bands of eumelanin on the feather (Figure 1b). *Pg* has been described as an incompletely dominant, autosomal gene located on chromosome 1 in close linkage with *Db* and *Melanotic* (*Ml*) (Carefoot, 1999; Moore & Smyth, 1972).

There are two distinct types of barring patterns in chicken, autosomal barring, and sex-linked barring. While sex-linked barring is adding a white bar on a pigmented background, autosomal barring is adding a black bar on a gold or silver background on individual feathers (Smyth Jr, 1990). Sex-linked barring is caused by the combined effect of regulatory mutation(s) and missense mutations in the tumor suppressor gene *CDKN2A* (Hellström et al., 2010; Schwochow Thalmann et al., 2017). Autosomal barring on the other hand has been suggested to be caused by the combined effect of the three autosomal loci *Db*, *E*, and *Pg* (Andersson et al., 2020; Carefoot, 1984,^1999^; Moore & Smyth, 1972) and is the trademark of some chicken breeds such as Fayoumi, Campine, and Westfälische Totleger.

*MC1R* is a key locus in pigmentation biology, and *MC1R* mutations are underlying pigmentation polymorphisms in many vertebrates. It codes for a G-protein-coupled receptor located in the plasma membrane of melanocytes. Upon activation by its agonist α-melanocyte-stimulating hormone (αMSH), MC1R goes through a conformation change triggering a signaling cascade, which eventually leads to an increase of intracellular cAMP levels, which in turn activates transcription of genes involved in pigment production. MC1R signaling promotes production of black/brown pigment (eumelanin), whereas low or no activity leads to the default production of red pigment (pheomelanin) (Garcia-Borron et al., 2005). In birds, associations of *MC1R* variants with pigmentation traits have been found both in domesticated species (Davila et al., 2014; Kerje et al., 2003; Nadeau et al., 2006; Ran et al., 2016; Takeuchi et al., 1996) as well as in wild birds (Baiao & Parker, 2012; Johnson et al., 2012; Lamichhaney et al., 2016; Mundy et al., 2004; San-Jose et al., 2015; Theron et al., 2001). There are at least six phenotypically distinct *Extension/MC1R* alleles described in chicken (Andersson et al., 2020), which are here listed according to dominance and decreasing amount of eumelanin: *Extended black* (E*E), Birchen (E*R), *wild-type* (E*N), brown (E*B), *buttercup* (E*BC), and *wheaten* (E*WH or E*Y depending on dominance). These alleles determine the distribution of black eumelanin across the body and *MC1R* is therefore considered to be involved in primary pattern formation (Andersson et al., 2020; Smyth Jr, 1990). Birds carrying the dominant *E* and *R* alleles usually exhibit very dark to black adult plumage while the remaining alleles create patterns of brown, salmon and wheat color in different body regions. In adult males, only two alleles (*E* and *R*) can be distinguished from wild-type, while the other variant alleles only cause distinct phenotypic effects in female plumage (Andersson et al., 2020; Smyth Jr, 1990).

In the present study, we generated an informative backcross involving the Fayoumi breed showing autosomal barring (expected genotype *Pg/Pg*, *Db/Db*, and *R/R* at the *E/MC1R* locus) and an in-bred line of Light Brown Leghorn (LBL) showing no feather pattern and assumed to be *wild-type* at all the major loci known to influence plumage color. We used pooled sequencing to map the loci affecting feather pigmentation patterns. Our data show that the major locus controlling the segregation of autosomal barring in this cross is located at the distal end of chromosome 11 where the *E/MC1R* locus is located. We also show that *Db* is not required for autosomal barring but that it affects the manifestation of this phenotype, and we did not find any evidence for the segregation at the proposed *Pg* locus.

## 2 | MATERIALS AND METHODS

### 2.1 | Animals

The Fayoumi population used in this study has been kept at an experimental farm over several decades and has shown none or little deviation from the characteristic pigmentation phenotype described for this breed. Feather samples and phenotype data were obtained from a Fayoumi backcross which was produced at the PEAT Poultry Experimental Facility (INRAE, Nouzilly; https://doi.org/10.15454/1.5572326250887292E12). Five silver autosomal barred Fayoumi dams (presumed genotype: *Pg/Pg*, *Db/Db*, *S/W*) were crossed with two Light Brown Leghorn males (*wild-type* genotype for all feather color loci to the best of our knowledge). From the F_1_ generation, twelve gold autosomal barred females (presumed genotype *Pg/pg*+, *Db/db*+, *s*+/*W*) were selected and crossed with another Light Brown Leghorn male to generate 365 backcross progenies in three batches with complete genotype and phenotype data, except that sex recordings were missing for two individuals. The chicks were phenotyped at hatch and at 12 weeks of age as well as genotyped for *Db*/*SOX10* and the E*R(*Fay*) allele. This protocol received the permit 02410.2 delivered by the French authority for animal experiments, after advice from the INRA Val de Loire ethical committee for animal experimentation.

Feather samples for gene expression were collected after sacrificing the animals, using electronarcosis followed by bleeding. The feather shafts were frozen in liquid nitrogen and stored at −80°C until further processing. DNA samples for genotyping were obtained from blood samples. Blood samples were taken from the wing vein and submerged with EDTA as an anti-clotting agent. Blood samples from other breeds showing various patterning phenotypes were obtained from fancy breeders.

### 2.2 | Whole-genome resequencing of the Fayoumi backcross

For the Fayoumi backcross, between 71 and 102 DNA samples per phenotype category were pooled in equimolar quantities and sequenced to 45× coverage on Illumina HiSeq2500 with 2 × 125 paired-end reads.

Sequencing adapters and low-quality bases were removed using *Trim Galore!* (with “--stringency 6 -q 15”). The trimmed reads were aligned to the chicken reference genome (GRCg6a/Galgal6) using *bwa mem* with the “-M” option. PCR duplicates were identified using *Picard* *MarkDuplicates*, and variant calling was done using the *GATK* toolkit (McKenna et al., 2010) v4.1.1.0. First, base quality scores were recalibrated using known variants in dbSNP150. Next, the *HaplotypeCaller* was used to call variants in gVCF format. All gVCF files were combined and the raw variants were scored and filtered by the *VariantRecalibrator* module using dbSNP150 as a training set (--truth-sensitivity-filter-level 90.0), followed by a hard filter which excluded SNPs with QD <2.0, FS >60.0, MQ <40.0, MQRankSum < −12.5, or ReadPosRankSum < −8.0 and indels with QD <2.0, FS >200.0 or ReadPosRankSum < −20.0.

Variants used in the linkage analysis were selected based on an earlier version of the analysis, which was performed as described above with the following differences: alignments were done against Galgal4 using *bwa aln* followed by *bwa sampe,* the *gatk* toolkit v3.2.2 was used, an *IndelRealigner* step was included, all recalibration steps were done using dbSNP140, and “--ts_filter_level 95.0” was used for the *VariantRecalibrator*. All genomic coordinates from this analysis have been updated to Galgal6 using the liftOver tool.

### 2.3 | Calculation of the fixation index (*F_ST_*)

*F*_*ST*_ was estimated for each called variant as $F_{ST}=\left(\pi_{between}-\pi_{within}\right)/\pi_{between}.$ values were then averaged across 30 kb sliding windows and visualized as a Manhattan plot after excluding regions with a very low number of variants. The scores for each region were transformed to a Z score, and regions with a score corresponding to *p* < .05 after Bonferroni correction were reported.

### 2.4 | Genotyping and Sanger sequencing of *MC1R* and *Db*/SOX10

The entire Fayoumi pedigree was genotyped for *Db*, an 8.4 kb deletion upstream of *SOX10*, by using primers and PCR conditions described elsewhere (Gunnarsson et al., 2011). The *MC1R* genotype was first evaluated by using an allele discrimination assay on a 7900 HT Fast Real-Time PCR System machine (LifeTechnologies). In brief, 10 ng of DNA was amplified in a reaction using 1× TaqMan Universal PCR mastermix (LifeTechnologies) with 1× Assay mix containing probe and primers and filled up with water to a total reaction volume of 5 μl/sample. The reaction mix was subjected to 40 cycles as follows: 15 s at 92°C and 1 min at 60°C, preceded by 1 × 10 min at 95°C. The data were analyzed using the software sds 2.3. Custom-made probes for the L133Q mutation were obtained from Thermo Fisher.

In order to select genetic markers for fine mapping, we used the resequencing data and extracted SNPs located between position 40,155,407 and 59,843,680 bp on chromosome 1, which occurred at a frequency of 0.4 to 0.6 in the “autosomal barring” pool and <0.2 frequency in the “wild-type” pool. Each SNP had to be covered by at least 20 reads. The same procedure was used for retrieving SNPs from chromosome 11 located between 15,539,481 and 19,932,279. Some high *F_ST_* SNPs (*F_ST_* > 0.35) between 6,258,804 bp and 71,048,324 on chromosome 2 were also included. The final composition of SNPs was chosen randomly by keeping the distance between neighboring SNPs in the range 189–322 kb on chromosomes 1 and 11. For chromosome 2, the SNPs were spread out across the chromosome, as there was no distinct haplotype. The genotyping service was provided by Neogen Europe Ltd, Geneseek. The haplotype composition was evaluated using Excel.

### 2.5 | Linkage analysis

Linkage analysis was performed using the cri-map 2.504 software and 76 SNPs obtained through the custom-made genotyping assay as well as genotype data at *MC1R* and *Db*/*SOX10* (Tables S2 and S3). The initial analyses were carried out using the entire pedigree. Genetic studies have predicted *Pg* to be located on chromosome 1 (Carefoot, 1999; Moore & Smyth, 1972), but evaluation of phenotype and genotype at *Db* in our cross suggested that *Db* is not required for autosomal barring. We therefore eliminated backcross individuals with wild-type plumage and only used those that showed pattern (autosomal barring or unclear) and carried the Fayoumi allele at *MC1R* as those could reveal a potential-associated locus on chromosome 1 if there was any. We also noticed a strong sex bias within the phenotype group “unclear” (Table 2), which was dominated by females. We therefore performed part of the analysis by only using E*R(*Fay*)/E*N backcross females.

### 2.6 | Expression analysis and allelic imbalance

RNA extraction from growing feather follicles, primer design, primer testing, gene expression assays as well as allelic imbalance testing was performed as described (Schwochow Thalmann et al., 2017). All primers used for expression analysis are provided in Table S10. Relative gene expression levels of target genes *MC1R*, *NQO1*, *CDH1*, and *WWP2* were calculated using the ΔΔCt method and normalized with two housekeeping genes: eukaryotic translation elongation factor (EEF2) and *β-actin* (Schwochow Thalmann et al., 2017). The following sequence variants were used for the allelic imbalance assays: *MC1R* (located at chr11:18,841,043), *NQO1* (T to C change; chr11:19,037,574), and *CDH1* (A to T change; chr11:18,874,575).

### 2.7 | Immunohistochemistry and in situ hybridization

For section immunostaining and in situ hybridization, fixed skin tissue was embedded in paraffin and sectioned at 6–7 μm. After de-paraffination, sections were processed for immunohistochemistry or in situ hybridization. The MITF antibody was from Abcam (ab12039, 1:200 dilution). The peroxidase staining was used after primary antibody treatment as described (Jiang & Chuong, 1992). Non-radioactive in situ hybridization was performed as described (Chuong et al., 1996). Briefly, the sections were treated with proteinase K (10 μg/ml in PBS) for 20 min, re-fixed with 0.2% glutaraldehyde/4% paraformaldehyde, and rinsed with PBT. The sections were then prehybridized in hybridization buffer (containing 50% formamide, 5× sodium citrate/sodium chloride buffer, 1% sodium dodecyl sulfate, 50 μg/ml heparin, 50 μg/ml tRNA) at 65°C for 1 hr. After prehybridization, sections were placed in new prehybridization buffer containing 1–3 μg/ml digoxigenin-labeled riboprobes and hybridized overnight at 65°C. Finally, sections were incubated with alkaline phosphatase-conjugated anti-digoxigenin Fab (Roche, Indianapolis, IN) overnight. Positive signals were detected by incubating the specimens with NBT (nitro-blue tetrazolium)/BCIP (5-b romo-4-chloro-3′-indolyphosphate) substrates (Promega, Madison).

### 2.8 | Whole-genome resequencing of chicken pools representing different within-feather patterning phenotypes

Six samples with pooled DNA from five individuals each of the Brahma, Buttercup, Fayoumi, Hamburg, Plymouth Rock, and Sebright breeds were sequenced to about 10× coverage on Illumina HiSeq 2,500 with 2 × 100 bp paired-end reads. Genome alignment and variant calling were done against Galgal6 using *bwa mem*. Variants were called with *HaplotypeCaller* from the gatk toolkit (v3.8) followed by filtering using the *VariantRecalibrator* and hard filters as described above.

Pooled heterozygosity was calculated as described (Rubin et al., 2010). For each observed SNP, the read count of the major allele, *n*_*MAJ*_, and the minor allele, *n_MIN_*, was calculated across all breeds. A pooled heterozygosity, *H_p_*, was calculated as $H_{p}=2^{*}\Sigma \left(n_{MIN}\right)/{\left(\Sigma \left(n_{MAJ}\right)+\Sigma \left(n_{MIN}\right)\right)}^{2}$ across all variants within a 30 kb window and then transformed to a Z score according to $ZH_{p}=\left(H_{p}-\mu \right)/\sigma .$ Negative Z-scores correspond to regions with less than expected heterozygosity, indicating that they may be identical by descent (IDB). Windows with Z-scores less than −4.70 were considered significant, which correspond to a *p*-value less than .05 after Bonferroni correction. The resulting Z score was visualized in Manhattan plot-style, omitting windows with too few variants.

For the visualization of all haplotypes near *MC1R* (Figure 5), we extracted all SNPs (including low-confidence ones) ±5kb (*n* = 105) or ±50kb (*n* = 1,424) of the *MC1R* gene.

## 3 | RESULTS

### 3.1 | Segregation of plumage color in a Fayoumi backcross population

**FIGURE 2:**
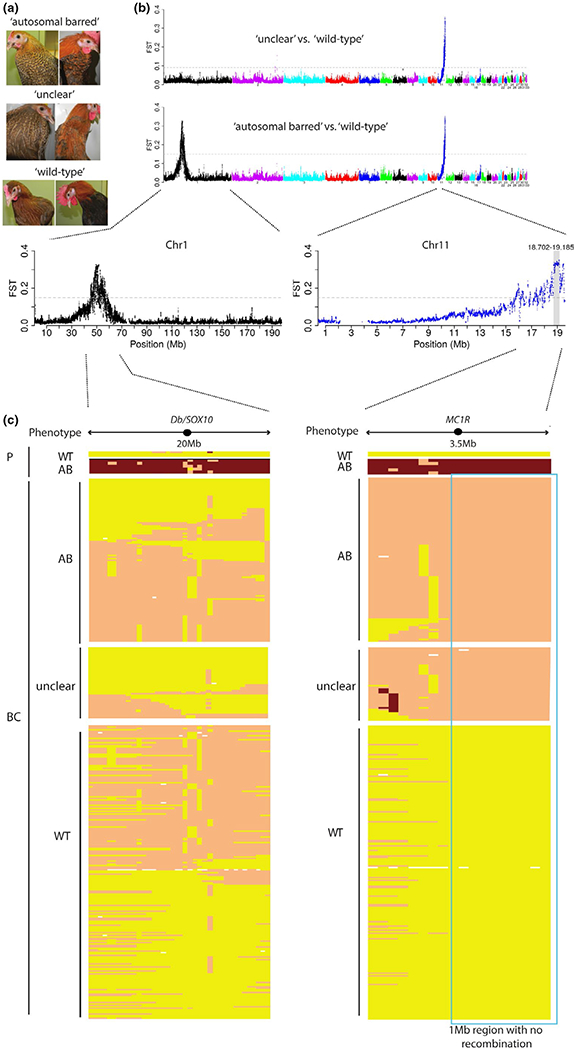
A 1 Mb region on chromosome 11 is associated with autosomal barring. (a) Segregation of pigmentation phenotypes among backcross progeny. Photograph credit: David Gourichon, INRA. (b) Genome-wide screen for genetic differentiation between three different pools (“autosomal barred,” “unclear” and “wild-type”) of Fayoumi/Light Brown Leghorn backcross offspring using *F*_ST_ values in 30 kb sliding windows. (c) SNP scoring within 20 Mb and 3.5 Mb regions on chromosomes 1 and 11, respectively, in parents (P) and offspring progeny (BC) showing autosomal barring (AB), “unclear” patterning or wild-type plumage. The positions of *MC1R* and *SOX10* are indicated. Please note that a higher density of SNPs was used toward the end of chromosome 11 visually suggesting that the 1 Mb interval is taking up half of the 3.5 Mb region. The borders of the 1 Mb non-recombining interval are based on a single recombinant. Yellow color indicates homozygosity for an allele inherited from the wild-type Light Brown Leghorn parental (*WT*), whereas red indicates homozygosity for a Fayoumi-derived allele. Orange color indicates heterozygosity. The panel for chromosome 11 represents 18 SNPs with the first situated at 15.8 Mb and the last one at 19.7 Mb. Forty-six SNPs were placed within the 20 Mb region on chromosome 1 with the first SNP located at 40 Mb and the last at 59.8 Mb. White fields indicate missing genotypes.

We crossed five Fayoumi females (presumed genotype *Pg*/*Pg*, *Db/Db*) displaying the typical black and white autosomal barring pattern with two Light Brown Leghorn males showing no patterning (*wild-type* at the *Pg* and *Db* loci) (Figure 1a). Twelve F_1_ females (heterozygous carriers of *Pg* and *Db*) showing the typical autosomal barring pattern of the Fayoumi breed were backcrossed with another Light Brown Leghorn male generating a total of 365 backcross progeny with complete phenotype information that were used for the genetic analysis. Of these 365, 102 (43 males and 59 females) chickens exhibited the typical autosomal barring pattern (AB), whereas 203 (99 males, 102 females, and 2 unrecorded sex) were classified as wild type (WT) for patterning (Table 1, Figure S2). A total of 60 birds were more difficult to categorize, 28 (9 males and 19 females) were neither plain nor did they have the typical autosomal barring pattern and were classified as “unclear” (Figure 2a). Furthermore, for 21 males initially phenotyped as AB at hatch and 7, which exhibited an irregular pattern, the pattern had completely disappeared at 12 weeks of age (Table S1, Figure S2), and instead, they developed a reddish taint in the belly region and were termed “red belly” (RB). This group also includes four males, which were scored as wild-type at hatch but developed the red belly phenotype by 12 weeks. These 28 + 4 male progenies classified as red belly were excluded from most analyses.

According to the assumed inheritance pattern of autosomal barring, we expected that 25%–50% of the backcross progeny should be heterozygous carriers of *Pg* and *Db* and show this phenotype, the reason for this range in expected frequency is because *Pg* has been reported to be linked to *Db* but no precise estimate of the recombination rate is available. We observed 102 AB versus 201 WT, 28 birds with unclear phenotype classification, and 32 with the red belly phenotype (Table 1). By genotyping, we confirmed homozygosity for the causal mutation at *Db/SOX10* (Gunnarsson et al., 2011) in all Fayoumi founder females (*Db*/*Db*) and all F_1_ females were heterozygous *Db*/*N* as expected. Surprisingly, not all of the autosomal barred backcross progeny were genotyped as carriers of *Db* based on the presence of the deletion upstream of *SOX10* (66 heterozygous carriers of *Db* and 36 non-carriers) whereas the “unclear” group was almost entirely (27 out of 32 chickens) *wild-type* for the *SOX10* deletion (Table 2). The WT group contained an almost equal distribution with 100 heterozygous carriers of *Db* and 103 non-carriers. Thus, the segregation and genotyping data question the critical role of the *SOX10* deletion for autosomal barring as well as the linkage between *Pg* and *Db*.

### 3.2 | A major locus underlying autosomal barring maps to a region on chromosome 11

Based on the phenotype data and the genotype data at the *Db*/*SOX10* locus described above, we set up four pools for whole-genome resequencing using Illumina HiSeq technology in an attempt to map the *Pg* locus. We used two different wild-type pools but with different genotypes at *Db*/*SOX10* (heterozygous carrier of *Db*, *n* = *99* or *wild-type*, *n* = 102) whereas the two other pools either contained clearly autosomal barred chickens (heterozygous carrier of *Db*, *n* = 71) or chickens with a less clear barring phenotype including all “unclear” progenies as well as some with a less pronounced autosomal barring pattern (*wild-type* at *Db*, *n* = 72). The pools were constructed taking into account the genotype at the *Db*/*SOX10* locus in order to maximize the chance to detect other loci affecting the patterning phenotype. All four pools were compared to each other, and regions of high differentiation between pools were determined using the Fixation index (*F*_*ST*_) in 30 kb sliding windows (Figure 2b; Figure S3). Depending on which pools were compared, two regions with high *F*_*ST*_, one on chromosome 1 and one on chromosome 11, were detected. The signal on chromosome 1 encompassing the *Db*/*SOX10* locus was expected since we constructed the pools on the basis of the genotypes at this locus. The signal on chromosome 11 covered the region 18.399–19.527 Mb (near the chromosome end) with the highest signal around 18.702–19.185 Mb, which included *MC1R* located at 18,840,646–18,841,590 bp. The signal on chromosome 11 was detected both when the “autosomal barring” pool and the “unclear” pool were compared with either one or both of the wild-type pools (Figure 2b).

### 3.3 | Fine mapping revealed a region of low recombination on chromosome 11

We used our resequencing data to identify a total of 100 highly informative SNPs. The SNPs were selected from the candidate regions on chromosome 1 (*n* = 63) and chromosome 11 (*n* = 21), and in addition a few relatively high *F_ST_* SNPs from chromosome 2 (*n* = 16) (Table S2). The entire pedigree was genotyped for these markers to validate the observed associations and improve the map resolution on chromosomes 1 and 11 (Table S3). A total of 46 SNPs on chromosome 1 were informative and revealed no haplotype that was shared among all individuals of either phenotype category (Figure 2c). However, a large proportion of the backcross progeny showing autosomal barring carried the *Db* haplotype whereas only three that were classified as unclear carried this haplotype revealing a clear association to a patterning phenotype. For chromosome 11, 20 SNPs were highly informative and revealed a region of 1 Mb without any recombination starting from about 18.8 Mb to the end of chromosome 11 (Figure 2c, Table S3). All birds phenotypically classified as autosomal barred or unclear were heterozygous for the *MC1R* haplotype (E*R(*Fay*)) inherited from the Fayoumi founder females. Linkage analysis revealed highly significant LOD scores for loci located at the distal end of chromosome 11 (LOD scores = 28.4–69.1), whereas only weak associations between autosomal barring and some markers on chromosome 1 were observed, irrespective of which subset of backcross progeny was used (Tables S4–S6).

The 1 Mb interval on chromosome 11 showing a complete association with patterning contains 29 genes including *MC1R* (Table S7). We used our resequencing data to extract SNPs that differed between autosomal barred and wild-type backcross progeny within the non-recombining region. The L133Q mutation was not extracted, most likely because it did not meet the coverage requirements of at least 20 reads/SNP. The obtained variant SNPs were analyzed using the online tool UCSC Variant Annotation Integrator (VAI), which resulted in a total of 6,627 variants. As expected, the great majority of those variants were located in non-coding regions such as intergenic regions (2,342), introns (2,298) as well as down- or upstream of genes (967 and 852, respectively; Figure S4), 19 variants were detected as potential splice variants and 149 as exonic variants. Of the 149 exonic variants, 107 were synonymous while 41 were non-synonymous (Figure S4). Ten of the genes in the interval showing no recombination harbored non-synonymous changes (Table S8). To further predict the possible effect of the missense mutations on protein function, we used PROVEAN (http://provean.jcvi.org/index.php), an online tool, which generates a score indicating how likely it is that a missense mutation affects protein function (Choi & Chan, 2015; Choi et al., 2012). Except for the known L133Q mutation in the *MC1R* E*R(*Fay*) allele, none of the other missense mutations associated with the *Fayoumi* haplotype from this region was predicted as having a deleterious effect on protein function (Table S8).

Thus, the screen for missense mutations within the 1 Mb region on chromosome 11 revealed only one missense mutation, the one in *MC1R*, that was predicted to affect protein function. Since *MC1R* is an obvious candidate gene for a pigmentation phenotype, we genotyped the entire pedigree for this missense (L133Q) mutation (Table 2). All chicken displaying autosomal barring (AB) or some kind of pattern (“unclear”) carried the variant allele at this position, whereas all plain (WT) chicken had the *wild-type* allele at *MC1R*. Even the 28 males that initially were phenotyped as AB but did not show any patterning at 12 weeks of age and were grouped into the red belly group carried the variant allele (Table S1). The segregation data at the *MC1R* locus among the backcross progeny deviated significantly from the expected 1:1 ratio (*χ*^2^ = 6.05, *df*= 1, *p* < .05). This is not specific for *MC1R* but applies to all markers in the 1 Mb region that did not recombine in this pedigree data.

To summarize, our genotyping data revealed a perfect association between a patterning phenotype and the variant E*R(*Fay*) allele at *MC1R*. It further revealed an incomplete association between the *Db* allele at *SOX10* and autosomal barring, which suggests that *Db* is not required to form the pattern but makes it more pronounced and easier to phenotype. There may be a third locus segregating in our cross, which is responsible for the red belly phenotype only present in males. Evaluating the genetic basis for this phenotype will be a subject for future studies.

### 3.4 | *MC1R*, *NQO1*, and *CDH1* are over-expressed in barred feathers but do not show allelic imbalance

**FIGURE 3:**
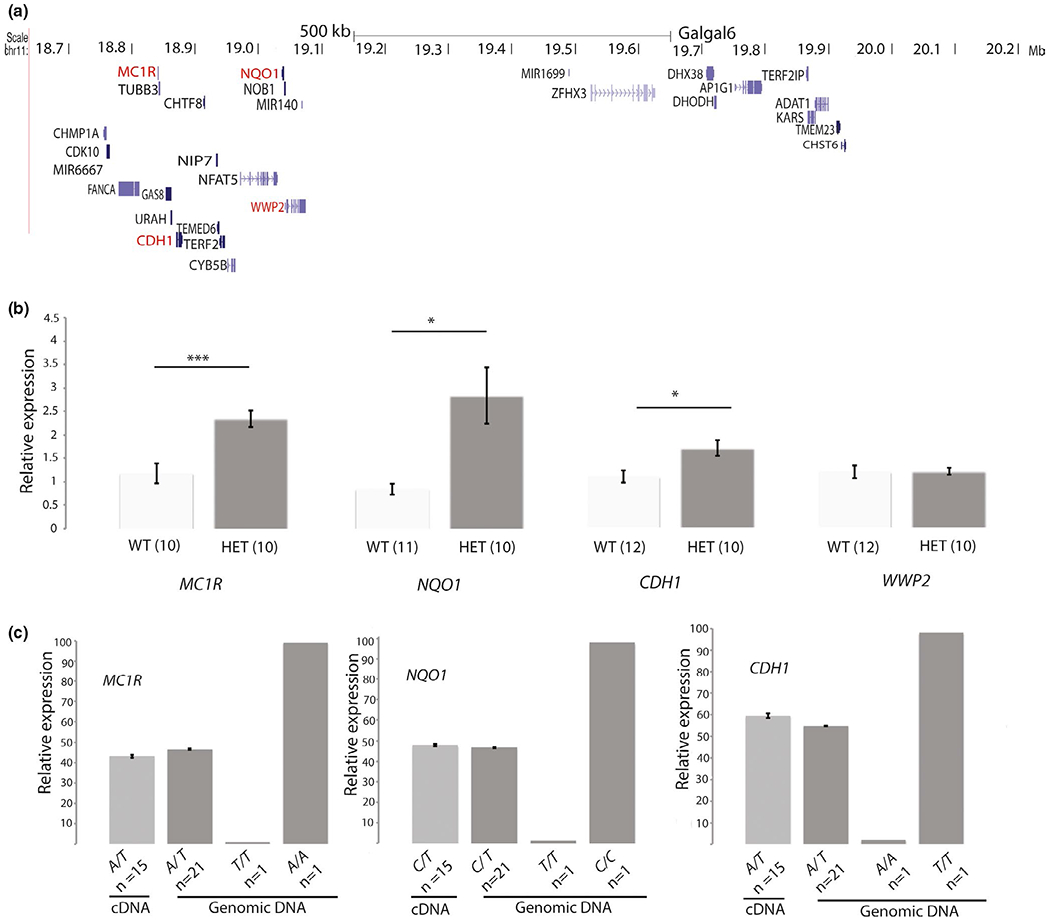
Expression analysis of candidate genes on chromosome 11. (a) Genes in the interval lacking recombination in the Fayoumi/Light Brown Leghorn cross. Positional candidate genes are highlighted with red text. (b) Relative gene expression levels of candidate genes on chromosome 11 in autosomal barred and non-barred feathers. *EEF2* and *βACTIN* were used as housekeeping genes. Significant differences are indicated by stars (Student’s *t* test; *p < .05, **p < .01, ***p < .001). (c) Relative proportion of the Fayoumi (*F*) allele at *MC1R*, *NQO1*, and *CDH1* in either cDNA samples from *F*/− feathers (light gray) or genomic DNA from different genotypes (dark gray; *F*/− , −/− and *F*/*F*)

We decided to explore possible regulatory changes in gene expression for genes located in the 1 Mb interval on chromosome 11 associated with patterning and with a putative role in melanocyte biology (Figure 3a; Table S7). In addition to the obvious positional candidate *MC1R*, we considered the *NAD(P)H* *Quinone Dehydrogenase 1* (*NQO1*) gene that codes for a cytoplasmatic 2-electron reductase, which is reducing quinones to hydroquinones and has been shown to affect the regulation of tyrosinase, thereby enhancing melanogenesis (Choi et al., 2010). We also examined the expression of *Cadherin 1* (*CDH1*), a calcium-dependent cell-cell adhesion protein, which among others, anchors melanocytes to surrounding keratinocytes (Vasioukhin et al., 2000) and is implicated in diseases such as vitiligo (Tarle et al., 2015). The last gene we evaluated is coding for an E3 ubiquitin-protein ligase (*ww* *domain-containing protein 1*, *WWP1*) involved in the tanning response following UV exposure in melanocytes (Cao et al., 2013). We measured the expression of these genes in growing chicken feathers from 10 to 12 non-barred and 10 autosomal barred birds. Three out of the four genes showed a statistically significant up-regulation of expression in barred feathers compared with non-barred feathers (Figure 3b): *MC1R* (fold change 2.3 ± 0.2, *p* = .0007, Student’s *t* test), *NQO1* (fold change 3.2 ± 0.7, *p* = .004) and *CDH1* (fold change 1.7 ± 0.2, *p* = .008). *WWP1* showed no statistically significant differential expression (*p* = .75, Student’s *t* test).

We next reasoned that if the elevated expression levels are the result of *cis*-regulatory changes, we expect to observe allelic imbalance in gene expression at one or more of the three genes (Figure 3c). We used SNPs in the transcripts to assess the relative expression of alleles and genomic DNA as control in which we expect a perfect 50:50 ratio. We used the T to A polymorphism causing the *MC1R*/E*R(*Fay*) allele (L133Q; chr11: 18,841,043 bp), and SNPs in *NQO1* (T to C change; chr11: 19,037,574 bp) and *CDH1* (A to T change; chr11:18,874,575 bp) to assess allelic expression in 15 heterozygous chickens utilizing cDNA from growing feathers and the pyrosequencing technology. The expression of the Fayoumi allele associated with each investigated gene was 43.1 ± 0.75% for *MC1R*, 47.7 ± 0.43% for *NQO1*, and 59.4 ± 1.11% for *CDH1*. These cDNA data and the genomic DNA control differed significantly from each other (*MC1R*: Student’s *t* test *p* = .0001; *NQO1*: Student’s *t* test *p* = .008; *CDH1*: Student’s *t* test *p* = 1 × 10^−6^), but these minor differences do not support a typical allelic imbalance expected in the presence of *cis*-regulatory effects, and we believe that they are unlikely to reflect actual expression differences with biological significance.

To summarize, *MC1R*, *NQO1*, and *CDH1* located within the non-recombining region show up-regulated expression in growing autosomal barred feathers but this up-regulation does not appear to be mediated by a *cis*-regulatory effect since none of the three genes shows clear allelic imbalance in favor of the Fayoumi allele. A possible explanation for this up-regulated expression is that the Fayoumi allele underlying autosomal barring results in a higher proliferation of melanocytes.

### 3.5 | The expression of MITF, ASIP, and KIT in Fayoumi feather follicles

**FIGURE 4:**
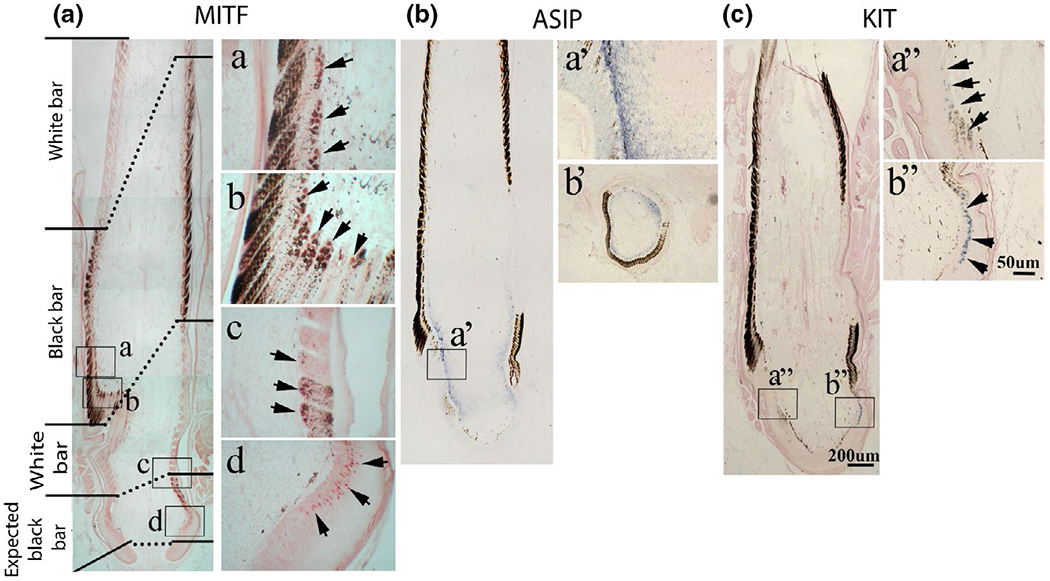
The expression of MITF, ASIP, and KIT in the Fayoumi feather follicle. (a) MITF immunostaining. Longitudinal feather sections with enlargement shown in the right column. a–c, black and white barred region. d, proximal follicle in collar bulge region. MITF-positive cells are seen in the basal layer of the feather filament epidermis in the stem cells region, in both black and white barb ridges. MITF-positive cells (red) are highlighted by box a, b, c, and d. (b) Longitudinal feather sections with ASIP in situ hybridization. ASIP is expressed in feather peripheral pulp facing non-pigmented bar regions (a’ and b’; b’ is a cross section). (c) Longitudinal feather sections with KIT in situ hybridization showing KIT-positive melanocyte stem cells at the follicle base near collar bulge region box a” and b”

Pigment bars can form due to the presence/absence of melanocytes or by differences in melanin production (eumelanin, pheomelanin, or no melanin) of the melanocytes that are present (Andersson et al., 2020; Lin et al., 2013). Autosomal barring is composed of alternating black and non-black bars (Figure 1). The non-black bars may be yellowish due to the presence of pheomelanin (Figure 1b) or more or less non-pigmented, like in Fayoumi chicken (Figure 1a) due to the presence of the *Silver* allele at *SLC25A2* that inhibits expression of pheomelanin (Gunnarsson et al., 2007). To characterize the mechanism underlying autosomal barring in Fayoumi chicken, we performed immunostaining of MITF (microphthalmia-associated transcription factor), a marker of melanocyte progenitor cells. MITF-positive cells are present in the proximal follicle where melanocyte stem cells are present, as shown by positive staining for KIT (Figure 4a,c). Toward the distal feather, barb branches start to form and pigment bars emerge. In both pigmented and non-pigmented regions, we observe the presence of MITF-positive melanocyte progenitors. This is particularly clear in the border region (Figure 4a,b). We explored what may repress the activity of these melanocyte progenitors in the non-black region. In Silver Laced Wyandotte chicken, ASIP was found to be present in the peripheral pulp facing the white region and suppress eumelanogenesis of MITF-positive progenitor cells (Inaba & Chuong, 2020; Lin et al., 2013). Using in situ hybridization, we detected expression of ASIP in the non-black regions. This is particularly clear in a cross section (Figure 4b). Thus, the non-black bars in autosomal barring of Fayoumi chicken are not caused by the absence of melanocytes, as seen in sex-linked barring (Lin et al., 2013; Schwochow Thalmann et al., 2017). Our results suggest that eumelanogenesis is suppressed due to the expression of ASIP in the non-black regions.

### 3.6 | Patterning phenotypes are strongly associated with certain *MC1R* (*E*) alleles

**FIGURE 5:**
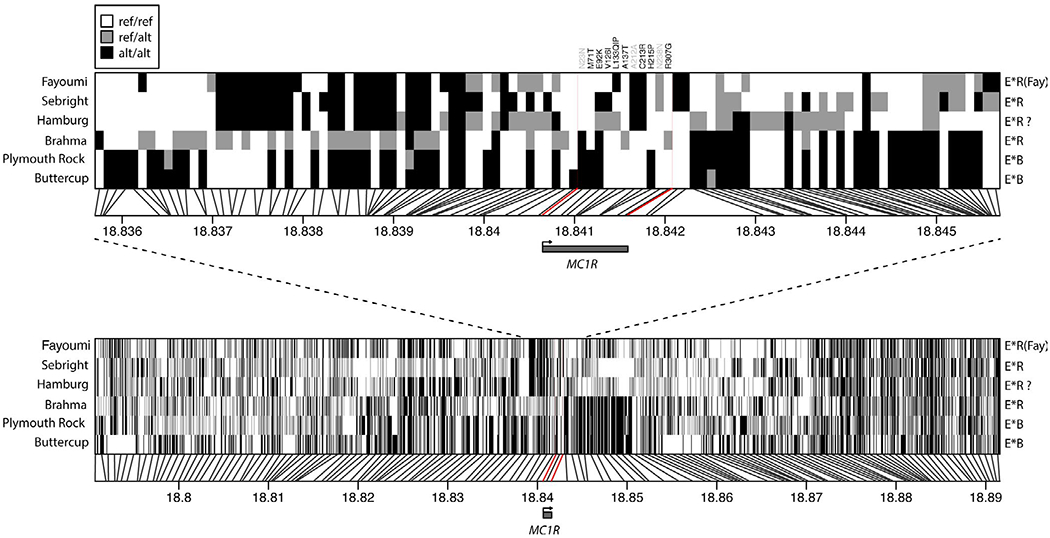
Region near *MC1R* reveals no shared haplotype associated with patterning. Predicted variants within ±5kb (top) or ±50kb (bottom) from the *MC1R* locus in Fayoumi and five other breeds (Brahma, Buttercup, Hamburg, Plymouth Rock, and Sebright) showing patterning phenotypes. The heatmaps show individual variants with vertical black lines indicating their position at the *MC1R*-flanking region on chromosome 11. Each breed was sequenced as a pool of five individuals and heterozygous positions thus represent a ~50/50 split within the pool. The *MC1R* coding sequence is indicated using red lines. The listed *MC1R* alleles use the following definitions: E*R(*Fay*) = 133Q, E*B = 71T-92K-215P, and E*R = 92K. The question mark for the Hamburg pool indicates that it was predicted to be variable for E92K, V126I, and L133P (note the P instead of Q) and thus is not fixed for E*R. Based on the data presented for Hamburg in Table S9, we deduce that the bird sequenced here carried the two following alleles: 92K-126I-133L (E*R) and 92E-126V-133P (E*?)

A number of chicken breeds are assumed to be fixed for the *Pg* allele based on the interpretation of the genetic basis for various plumage color variants (Figure 1b; Figure S1). We therefore performed whole-genome pooled sequencing of birds from six chicken breeds (*n* = 5 each), exhibiting a variety of pigmentation patterns: Partridge Plymouth Rock—penciling, Buttercup—autosomal barring, Silver Sebright—single lacing, Brahma—penciling, Hamburg—spangling, and Fayoumi—autosomal barring (Figure 1; Figure S1). This was carried out with two purposes (i) to explore whether all breeds predicted to be fixed for the *Pg* allele share any region of the genome that is identical by descent (IBD) and (ii) if these breeds share an IBD region within the 1 Mb region on chromosome 11 defined in our Fayoumi backcross population. Thus, the reason for using only five individuals per pool was that our aim was not to estimate allele frequencies, but to identify sequence variants that are fixed in these breeds since all individuals within the breeds show a patterning phenotype. The analysis of the pooled data revealed no striking IBD region shared by all populations neither within the chromosome 11 interval nor in the entire genome (Figure S5). However, the most striking finding was that all populations carried only two alleles *Birchen* (E*R or E*R(*Fay*)) or *brown* (E*B) at *MC1R*, while not sharing any common haplotype around *MC1R* (Figure 5).

A striking finding when comparing the 10 kb region harboring *MC1R* is the lack of linkage disequilibrium between sequence variants in this region (Figure 5), a total contrast to the lack of recombination over a 1 Mb region reported in this study based on pedigree analysis. This analysis also demonstrates that the *Birchen* (E*R) allele previously defined based on its phenotypic effect on plumage color is genetically heterogenous both as regards the *MC1R* coding sequence as well as the flanking sequences that may harbor regulatory variants affecting *MC1R* expression. For instance, the Brahma breed is considered fixed for the E*R allele but the Brahma birds included in this study are apparently segregating for at least two alleles both carrying the E92K mutation but differing as regards other missense mutations in *MC1R* as well as the haplotype upstream of the coding sequence (Figure 5).

Since there was no shared haplotype detected for breeds with a patterning phenotype at *MC1R* or elsewhere in the genome (Figure 5 and S5), but they all carry an *MC1R* allele with an activating mutation, we were considering the hypothesis that different *MC1R* alleles with similar functional effects are required for patterning phenotypes. Genotyping and subsequent sequencing of 60 Fayoumi chicken from different generations from the flock initially used for linkage mapping revealed that they are not fixed for E*R(*Fay*) (L133Q) but also segregate for the E*R allele (Table S9), which involves a missense mutation (E92K) resulting in constitutive activation (Benned-Jensen et al., 2011; Ling et al., 2003; Robbins et al., 1993) and known to be associated with melanism in other species (Baiao & Parker, 2012; Kerje et al., 2003; Mundy et al., 2004; Nadeau et al., 2006). Despite the segregation of two different *MC1R/E* alleles, there has been no obvious heterogeneity in the phenotypic appearance of autosomal barring in this Fayoumi line suggesting that the two alleles have a similar effect on patterning in this breed. We further extended our *MC1R* sequencing efforts to 16 additional individuals each from three of the *Pg* breeds (Partridge Plymouth Rock, Hamburg Silver Spangled and Sebright Silver) and found that all carried the E92K mutation (Table S9).

## 4 | DISCUSSION

The *Patterning* locus has been considered the major locus controlling within-feather pigmentation patterns in chickens (Figure 1b). Previous studies assigned this locus to chicken linkage group 3 (Carefoot, 1987; Moore & Smyth, 1972), now known to reside on chromosome 1. According to this model, we expected that the segregation of autosomal barring in our Fayoumi × Light Brown Leghorn backcross should be controlled by a locus on chromosome 1 in combination with the *Dark brown/SOX10* locus located on the same chromosome. However, the present study demonstrates that a locus located at the distal end of chromosome 11 is underlying autosomal barring in this pedigree. Our linkage data did not pinpoint a single gene associated with this phenotype due to the lack of recombination in a 1 Mb region. However, other data strongly suggest that autosomal barring in this pedigree is caused by the E*R(*Fay*) allele, characterized by the missense mutation L133Q, at the *MC1R* locus located in this interval. Pooled genome resequencing of six different breeds all exhibiting various types of patterning (previously assumed to be controlled by the *Patterning* locus) did not reveal any IBD region, which was expected if they were sharing the same causal mutation. However, the most striking feature was that all six breeds carried either *Birchen* (E*R) or *brown* (E*B) alleles at the *MC1R* locus. We observe that *MC1R* exhibits a high level of genetic diversity in its flanking sequences in addition to diversity within the coding sequence. We propose that *MC1R* is the major patterning locus in chickens; that is, different mutations causing altered regulation of MC1R signaling promote the development of feather patterns in interaction with other loci. One such interacting locus is definitely *Dark brown/SOX10*, others are melanotic (*Ml*) and Columbian restriction (*Co*) for which no underlying causal gene has been reported yet (Figure 1b). Furthermore, it is possible that regulatory mutations affecting *MC1R* expression also contribute to the complex inheritance of within-feather patterns in chicken as previously suggested (Ling et al., 2003).

One third of the autosomal barred backcross offspring were *wild-type* at the *SOX10* locus, demonstrating that the *Db* allele is not required for the autosomal barring phenotype. This is in contrast to previous reports in which *Db* was claimed to be required to exhibit autosomal barring in Fayoumi chicken (Carefoot, 1984,^1999^; Moore & Smyth, 1972). However, as almost all individuals carrying the causal *MC1R* allele but with an “unclear” feather pattern were *wild-type* at *Db*, we propose that the *Db* mutation is contributing to the regularity and clearness of autosomal barring, although not required for its formation. It is therefore likely that breeders specifically selected *Db* carriers for a more appealing phenotype, which may have led to the assumption that *Db* is required for autosomal barring. Thus, the genetic basis for patterning in chicken needs to be reconsidered since it is clear that there is not a universal pattering gene such as *Pg*, but that the *MC1R/E* locus plays a major role for patterning. However, the present study does not completely rule out the possibility of the existence of a *Patterning* locus in addition to *MC1R/E*, because it is possible, but unlikely, that the Fayoumi and Light Brown Leghorn founders used in the present study are all homozygous *Pg/Pg* despite the fact the latter do not show any patterning phenotype (Figure 1a). Nevertheless, our study provides strong evidence against the previous assumption that a *Patterning* locus on chromosome 1 has a predominant role for the presence/absence of within-feather patterns in chicken (Smyth Jr, 1990).

The presence of an activating *MC1R/E* mutation may be permissive but not sufficient to cause feather patterning because there are breeds carrying the activating E92K mutation but do not show patterning. In fact, none of the breeds examined in this study carried the top dominant allele *Extended Black* (*E*), which also has the E92K mutation and is associated with solid black colored feathers (Andersson et al., 2020). Thus, further pedigree experiments are required before we fully understand the genetic basis for the rich phenotypic diversity in within-feather patterning in this species (Figure 1 and S1).

We observed a surprisingly low rate of recombination in the 1 Mb interval at the distal end of chromosome 11 given the fact that the average recombination rate in chicken is about 4 cM/Mb. A possible explanation for a low rate of recombination is the presence of an inversion. However, it is unlikely that this is the case for this region because a similarly low rate of recombination has beem reported in previous studies (Groenen et al., 2009; Pengelly et al., 2016).

The expression data in feather follicles are consistent with our interpretation that autosomal barring is caused by an activating coding mutation at the *MC1R* locus. We documented up-regulated expression of *MC1R*, *CDH1*, and *NQO1* in autosomal barred feathers compared with wild-type feathers consistent with increased pigment production or an expansion of the number of pigment cells (Figure 4b). However, none of the three loci showed a clear allelic imbalance as expected if the causal mutation was a *cis*-acting regulatory mutation affecting the expression of one or more of these genes.

Ling et al. (2003) investigated the functional effects of different chicken *MC1R* alleles using transfection studies. They found that the E92K mutation present in the E*E, E*R, and E*B alleles is causing constitutive activation of the receptor. In contrast, they did not observe a similar effect for the L133Q mutation underlying the E*R(*Fay*) allele, which has been phenotypically assigned to the E*R group of *MC1R* alleles in chicken. However, these pharmacological studies carried out in mammalian cells do not perfectly replicate the endogenous conditions for chicken *MC1R*; for instance, important cofactors may be absent during the assay. Furthermore, these missense mutations may occur in strong linkage disequilibrium with regulatory mutations that affect MC1R expression. Thus, an important functional effect may not be manifested under these experimental conditions. In fact, the segregation of both the E92K and L133Q alleles in the line of Fayoumi chickens used in the present study without an obvious heterogeneity in phenotype shows that the two alleles must have similar effects on autosomal barring.

In birds, distinct pigment patterns can form across the body or within a feather (Inaba & Chuong, 2020). Horizontal barring in feathers is a striking pattern. Genetic studies of sex-linked barring established that this phenotype is caused by the combined effect of regulatory mutations and changes in the coding sequence of *CDKN2A* (Hellström et al., 2010; Schwochow Thalmann et al., 2017). Interestingly, MITF-positive cells are absent in the white barred regions, and there is no ASIP expression in the associated peripheral pulp of sex-linked barring feathers (Lin et al., 2013). Feathers are made from the distal to the proximal end (Chen et al., 2015). Careful analyses revealed that the depletion of melanocytes is considered to be based on CDKN2A-dependent precocious differentiation of melanocytes. When melanocyte stem cells sense the absence of melanocytes, another wave of melanocyte progenitor cells is produced to make the next black bar (Lin et al., 2013). In contrast, in the autosomal barring phenotype, we observed MITF-positive cells to be present in both the black and non-black barred regions during their formation. The pattern appears to be controlled by ASIP in the peripheral pulp region. How ASIP expression in the dermal cells is controlled is not known. In developing Japanese quail embryos, longitudinal pigmented stripes form on the dorsal trunk, and Japanese quail melanocytes transplanted to embryonic day 6 White Leghorn chicken embryos can induce ASIP in adjacent dermis (Inaba et al., 2019). Thus, we can speculate that melanocytes within the feather follicle at certain functional states may also induce ASIP expression.

Why is the presence of some *MC1R* mutations enhancing *MC1R* signaling and melanogenesis associated with within-feather pigmentation patterns as shown here, rather than just make feathers darker as predicted from the effects of *MC1R* mutations on hair pigmentation in mammals? Prum and Williamson (2002) proposed that within-feather pigmentation patterns might be caused by a Turing reaction-diffusion model. MC1R signaling stimulates melanogenesis, and it is possible that this, by an unknown mechanism, also induces ASIP expression in neighboring cells, and that ASIP in turn suppresses MC1R signaling as indicated in this study (Figure 4). This model gains some support from our finding that in addition to up-regulated expression of *MC1R*, also the closely linked *NQO1* and *CDH1* genes show higher expression in autosomal barred feathers; both genes have an established role in pigment cell biology (Choi et al., 2010; Vasioukhin et al., 2000). Thus, the presence of the E*R(*Fay*) allele results in more active melanocytes and/or an expansion of the number of melanocytes that may affect the interaction of different cell types that together determine within-feather pigmentation patterns. This model can be tested by gene editing and/or in vivo transfection experiment manipulating *MC1R* and *ASIP* expression.

The present study is also relevant for understanding the basis for camouflage color in birds. Stippling (Figure 1b) is a wild-type camouflage pattern typical for female birds nesting on the ground, and some juvenile birds present on the ground before they can fly, like gulls, terns and certain shorebirds. Our results support a role for ASIP-MC1R interactions in the formation of within-feather patterns, at least in some birds, and that increased MC1R signaling not only leads to a darker plumage but can also adjust the appearance of camouflage patterns. The importance of ASIP-MC1R interactions for camouflage patterns is also evident in mammals (Andersson, 2020). Dominant *MC1R* mutations causing constitutive activation as well as recessive mutations inactivating MC1R function disrupt the characteristic camouflage pattern in piglets (Kijas et al., 2001), which is also observed in chickens since the dorsal stripes of a wild-type day-old chick are not observed in fully black breeds, such as the Black Castellana, as well as in the fully red breeds, such as the Rhode Island Red. In the case of the Fayoumi, the day-old chicks show incomplete stripes on the back. Furthermore, ASIP-MC1R interactions are underlying winter white camouflage color in snow-shoe hares (Jones et al., 2018).

The present finding that genetic variation at *MC1R* is affecting pigmentation patterns in chicken is interesting in relation to reports that the MC1R antagonist ASIP has a role in feather pigmentation (Lin et al., 2013; Yoshihara et al., 2011; Zhang et al., 2013) and that *ASIP* is strongly associated with differences in throat pigmentation in golden-winged and blue-winged warblers (Toews et al., 2016) and a plumage trait in white wagtails (Semenov et al., 2021). These data together suggest that ASIP-MC1R interaction may play a pivotal role for pigmentation patterns in birds in general and that this interaction may have a greater impact in the growing feather that will develop into a two-dimension structure, as compared to the single dimension of the hair developing from mammalian hair follicles.

## Supplementary Material

Fig. S1

Fig. S3

Fig. S2

Fig. S5

Fig. S4

Table S1

Table S2

Table S4

Table S5

Table S6

Table S3

Table S7

Table S8

Table S9

Table S10

## Figures and Tables

**TABLE 1:** Segregation of four different phenotypes in the Fayoumi/Light Brown Leghorn backcross

Sex	Phenotype at 12 weeks of age				Total
	AB	WT	unclear	RB	
Males	43	99	9	32	183
Females	59	102	19	0	180
Unknown^a^	0	2	0	0	2
Total	102	203	28	32	365

Abbreviations: AB, autosomal barring; RB, red belly; unclear, irregular pattern; WT, wild-type.

a Sex recordings missing.

**TABLE 2:** Phenotype–genotype associations among backcross progenies of the Fayoumi/Light Brown Leghorn cross

Genotype		Phenotype				
		AB	WT	unclear	RB	Total
*SOX10*	Db*N/N	36	103	25	27	191
	Db*Db/N	66	100	3	5	174
*MC1R*	E*N/*N*	0	203	0	3	206
	E*R(*Fay*)/*N*	102	0	28	29	159
Total		102	203	28	32	**365**

Abbreviations: AB, autosomal barring; RB, red belly; unclear, irregular pattern; WT, wild-type.

## Data Availability

All sequencing data from this study are available through NCBI Sequence Read Archive BioProject PRJNA694957 (SAMN17582112, Autosomal barred pool; SAMN17582113, Dark brown (Db) pool; SAMN17582114, Wild-type pool; SAMN17582115, unclear pool) and PRJNA679787 (SAMN16846355, Sebright; SAMN16846356, Brahma; SAMN16846357, Hamburg; SAMN16846358, Plymouth_Rock; SAMN16846359, Buttercup; SAMN16846360, Fayoumi).

## Significance

Within-feather patterns are an important part of the stunning diversity of plumage color in birds. Barring is a common feather pigmentation pattern present, for instance, in hawks and owls. This study dissects the genetic basis for autosomal barring in the domestic chicken using pedigree analysis and molecular characterization. We show that the autosomal barring phenotype co-segregates with a missense mutation L133Q in the melanocortin 1 receptor (*MC1R*) gene. A general observation across breeds was that various feather pigmentation patterns in chicken are associated with missense mutations in the *MC1R* gene.
