# Supplementary figures and images for: The feather pattern autosomal barring in chicken is strongly associated with segregation at the MC1R locus
Source: Pigment Cell Melanoma Res. Author manuscript; Available in PMC 2022 Nov 1. (PMC8484376; doi:10.1111/pcmr.12975)

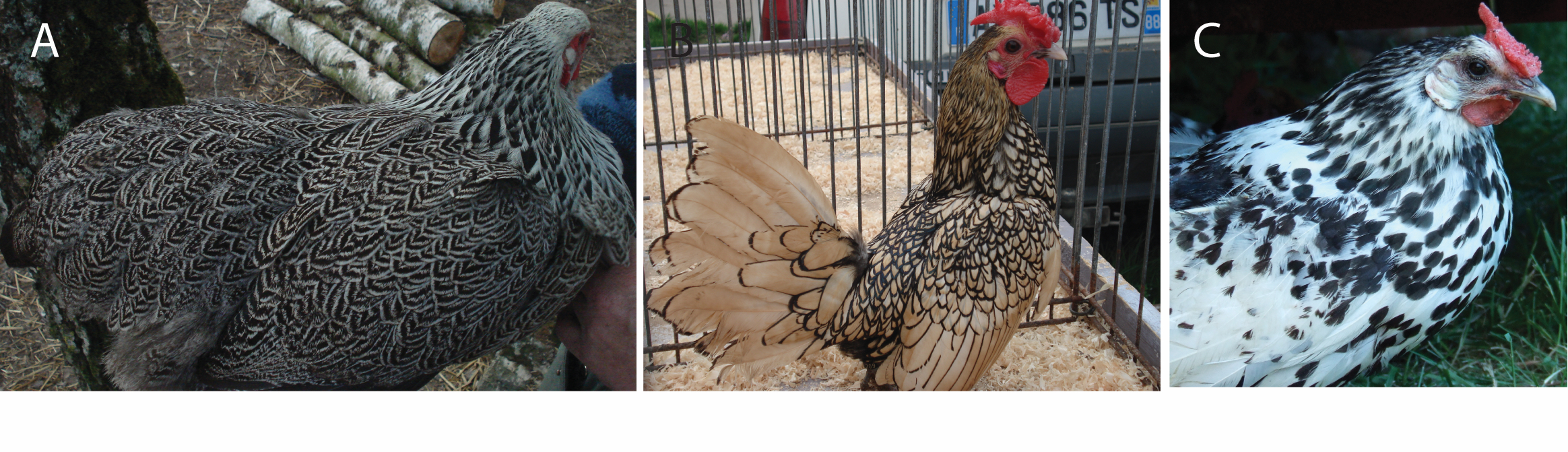

Supplement: Fig. S1 [file NIHMS1723557-supplement-Fig__S1.jpeg]

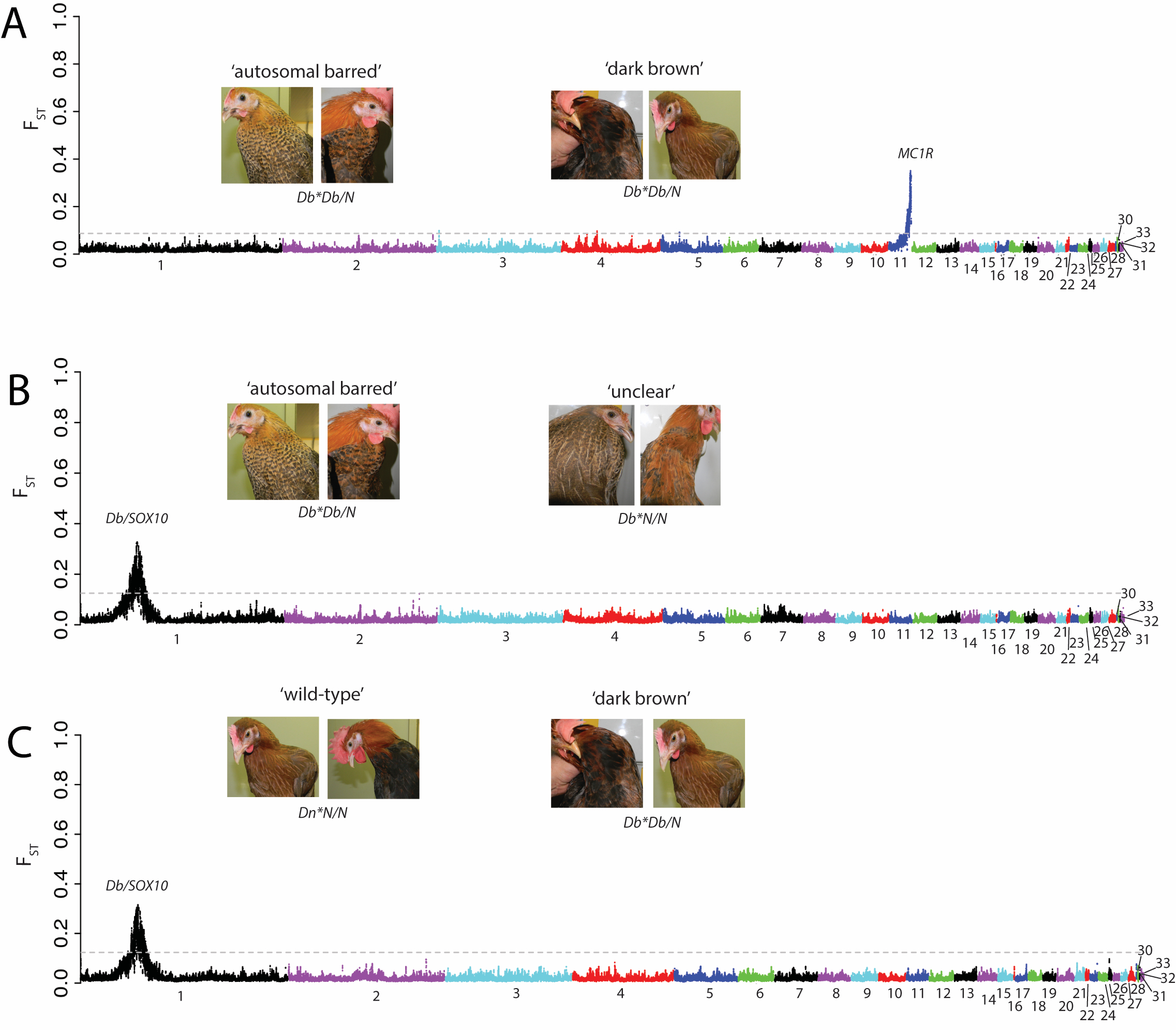

Supplement: Fig. S3 [file NIHMS1723557-supplement-Fig__S3.jpeg]

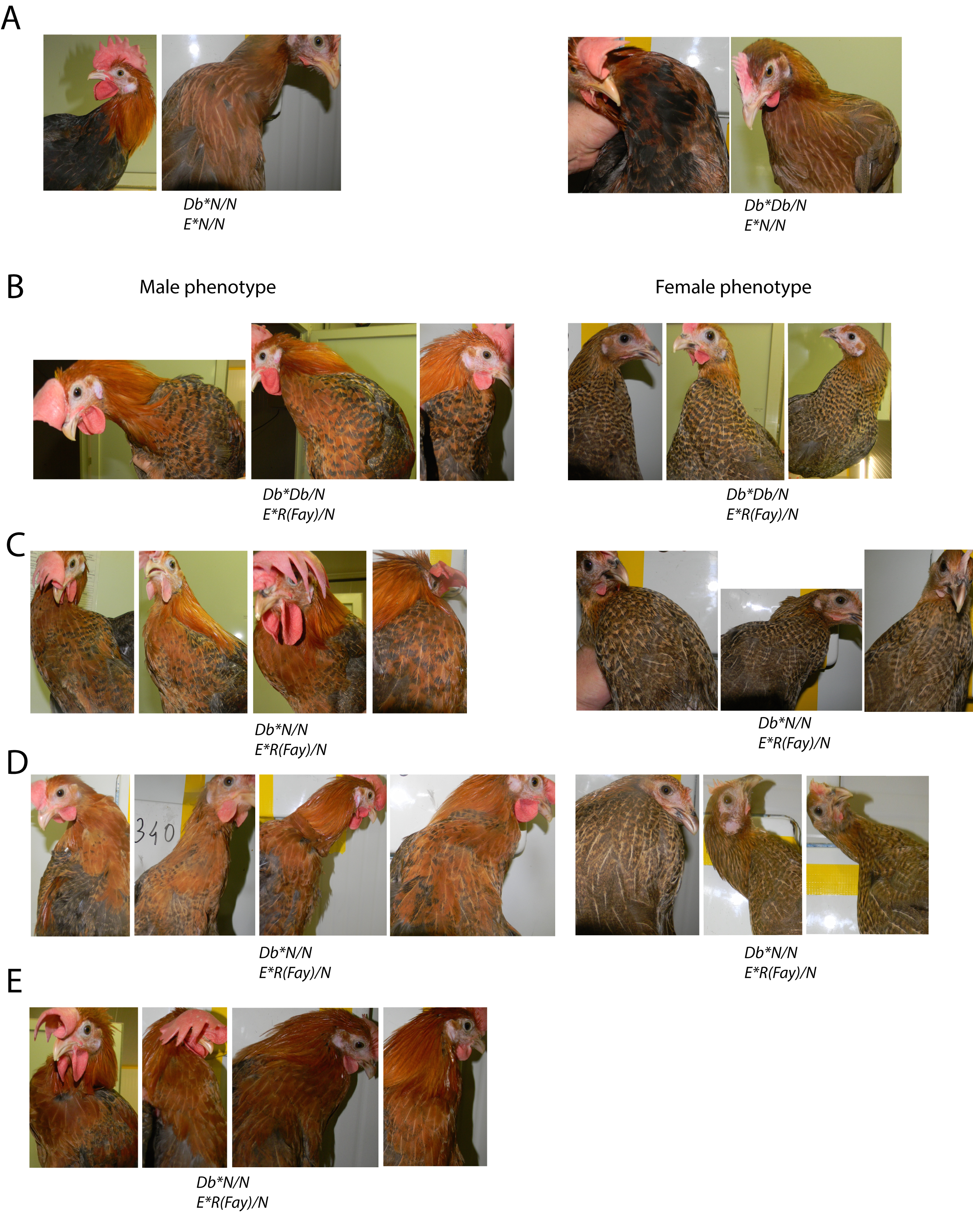

Supplement: Fig. S2 [file NIHMS1723557-supplement-Fig__S2.jpeg]

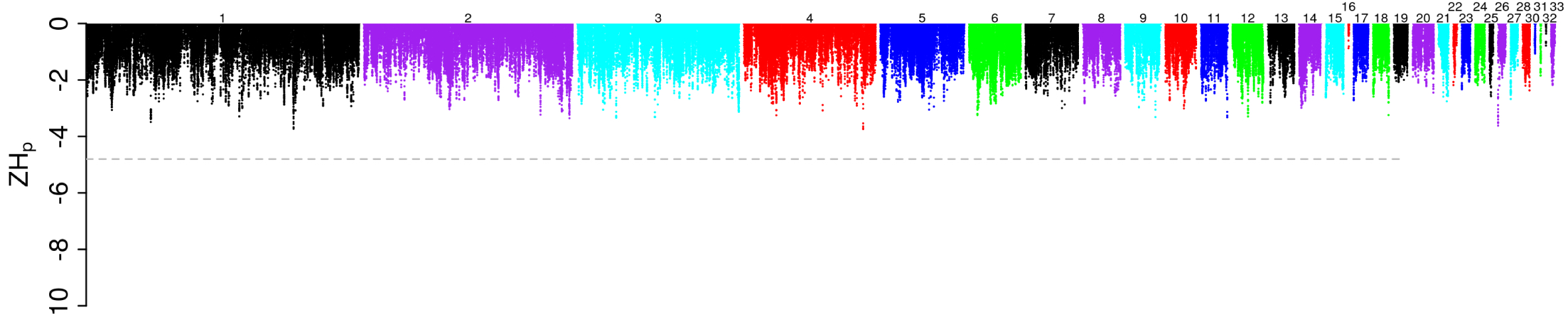

Supplement: Fig. S5 [file NIHMS1723557-supplement-Fig__S5.jpeg]

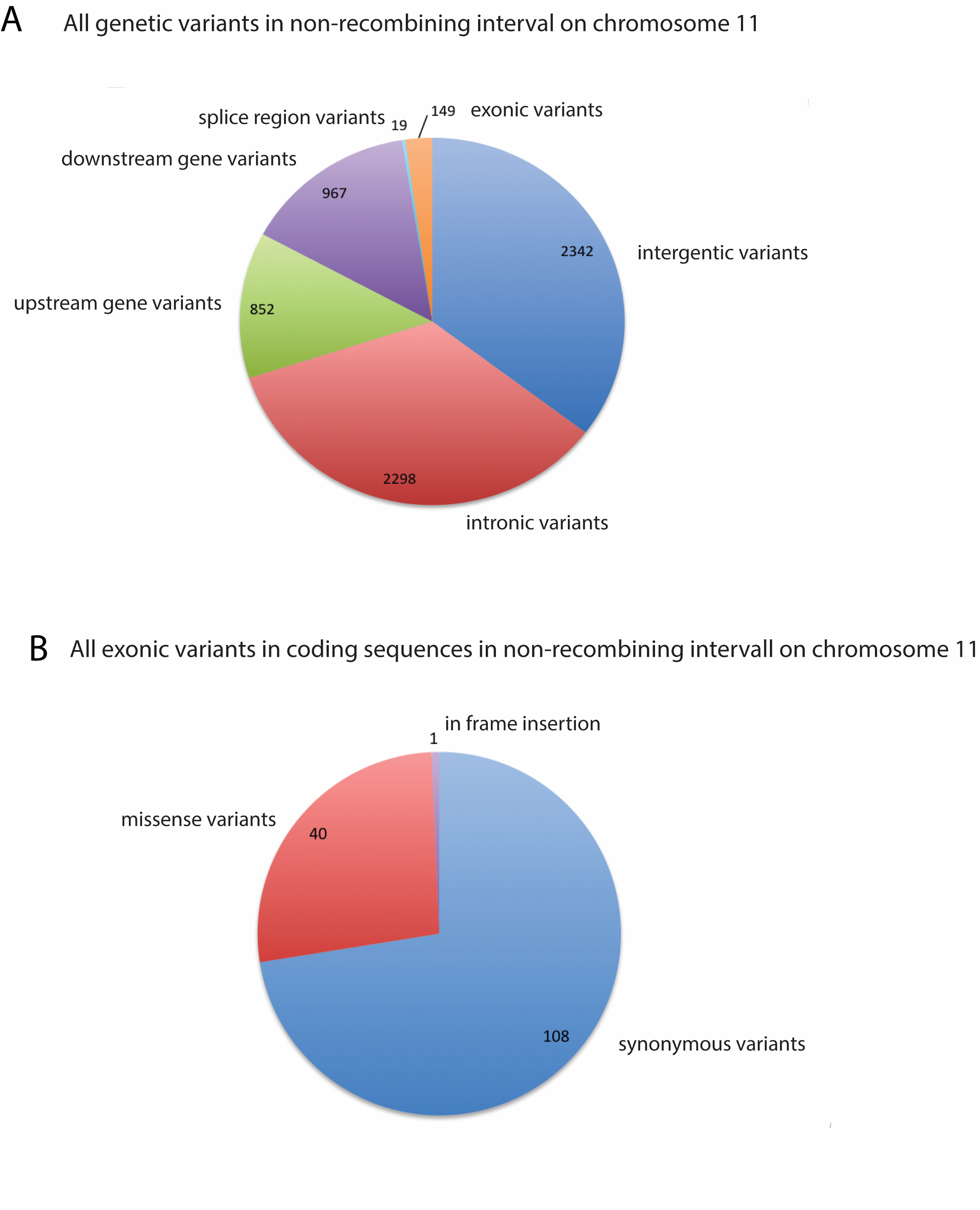

Supplement: Fig. S4 [file NIHMS1723557-supplement-Fig__S4.jpeg]
