# Supplementary material for: The feather pattern autosomal barring in chicken is strongly associated with segregation at the MC1R locus
Source: Pigment Cell Melanoma Res. Author manuscript; Available in PMC 2022 Nov 1. (PMC8484376; doi:10.1111/pcmr.12975)
Supplement: Table S1 [file NIHMS1723557-supplement-Table_S1.docx]

**Table** **S1.** Phenotype at hatch of backcross progenies classified as the red belly (RB) phenotype at 12 weeks of age. At older age the red belly phenotype masks the autosomal barring phenotype. AB=autosomal barring; WT=wild type.

|  | **Phenotype** | | | |  |
| --- | --- | --- | --- | --- | --- |
| **Age** | **AB** | **WT** | **unclear** | **RB** | **Total** |
| **At hatch** | 21 | 4 | 7 | 0 | 32 |
| **At 12 weeks** | 0 | 0 | 0 | 32 | 32 |
