# Supplementary material for: The feather pattern autosomal barring in chicken is strongly associated with segregation at the MC1R locus
Source: Pigment Cell Melanoma Res. Author manuscript; Available in PMC 2022 Nov 1. (PMC8484376; doi:10.1111/pcmr.12975)
Supplement: Table S2 [file NIHMS1723557-supplement-Table_S2.docx]

**Table** **S2.** Number of genetic markers on three different chromosomes used for linkage analysis.

| **Chromosome** | **# SNPs in the region** | **Region size (Mb)** | **Chosen SNPs** | **Average distance (kb)** | **Removed SNPs** | **Used for analysis** |
| --- | --- | --- | --- | --- | --- | --- |
| 1 | 18,619 | 17 | 63 | 322 | 17 | 46 + *Db* |
| 2 | 508 | 65 | 16 | 3,613 | 6 | 10 |
| 11 | 4,105 | 3 | 21 | 189 | 1 | 20 + *MC1R* |
