# Supplementary material for: The feather pattern autosomal barring in chicken is strongly associated with segregation at the MC1R locus
Source: Pigment Cell Melanoma Res. Author manuscript; Available in PMC 2022 Nov 1. (PMC8484376; doi:10.1111/pcmr.12975)
Supplement: Table S4 [file NIHMS1723557-supplement-Table_S4.docx]

**Table** **S4.** Linkage analysis of the phenotype autosomal barring against markers on chromosome 1. The entire pedigree has been used. All markers with maximum LOD score> 2.0 are listed.

| **Marker** | **Position Galgal4 (bp)** | **Position Galgal6 (bp)** | **Rec.fraction** | **LOD score** |
| --- | --- | --- | --- | --- |
| **M34** | 50457641 | 50600292 | 0.37 | 3.89 |
| **M40** | 51356204 | 51512638 | 0.39 | 2.49 |
| **M42** | 51694562 | 51848753 | 0.41 | 2.27 |
| **M48** | 53218396 | 53254833 | 0.41 | 2.02 |
| **M49** | 53533418 | 53571266 | 0.41 | 2.09 |
| **M51** | 54153352 | 54190564 | 0.41 | 2.18 |
| **M52** | 54555335 | 54395023 | 0.41 | 2.09 |
| **M54** | 55181657 | 55026114 | 0.40 | 2.87 |
| **M56** | 55914286 | 55762852 | 0.39 | 3.27 |
| **M58** | 56636567 | 56485406 | 0.40 | 2.77 |
| **M59** | 56972224 | 56821053 | 0.40 | 2.59 |
| **M61** | 58149062 | 57997786 | 0.41 | 2.02 |
| **M63** | 59607171 | 59464979 | 0.41 | 2.02 |
