# Supplementary material for: The feather pattern autosomal barring in chicken is strongly associated with segregation at the MC1R locus
Source: Pigment Cell Melanoma Res. Author manuscript; Available in PMC 2022 Nov 1. (PMC8484376; doi:10.1111/pcmr.12975)
Supplement: Table S5 [file NIHMS1723557-supplement-Table_S5.docx]

**Table S5.** Linkage analysis of the phenotype autosomal barring against markers on chromosome 1. Only backcross offspring with the variant allele at *MC1R* were used for analysis (*E*R(Fay****)****/E*N*) in order to detect regions contributing to the phenotype. All markers with maximum LOD score> 2.0 are listed.

| **Marker** | **Position Galgal4 (bp)** | **Position Galgal6 (bp)** | **Rec.fraction** | **LOD score** |
| --- | --- | --- | --- | --- |
| **M25** | 48355447 | 48498780 | 0.33 | 2.19 |
| **M26** | 48718888 | 48862557 | 0.33 | 2.32 |
| **M27** | 49022195 | 49165859 | 0.31 | 2.65 |
| **M28** | 49136914 | 49280579 | 0.31 | 2.65 |
| **M31** | 49801782 | 49280579 | 0.31 | 2.65 |
| **M32** | 50117333 | 50259829 | 0.31 | 2.65 |
| **M33** | 50263114 | 50405610 | 0.31 | 2.65 |
| **DB** | 50891000 | 51034258 | 0.34 | 2.02 |
| **M42** | 51694562 | 51848753 | 0.34 | 2.02 |
| **M44** | 52091920 | 52246126 | 0.34 | 2.02 |
| **M45** | 52325888 | 52480092 | 0.34 | 2.02 |
| **M54** | 55181657 | 55026114 | 0.34 | 2.02 |
| **M56** | 55914286 | 55762852 | 0.330 | 2.12 |
| **M58** | 56636567 | 56485406 | 0.30 | 3.00 |
| **M59** | 56972224 | 56821053 | 0.31 | 2.65 |
