# Supplementary material for: The feather pattern autosomal barring in chicken is strongly associated with segregation at the MC1R locus
Source: Pigment Cell Melanoma Res. Author manuscript; Available in PMC 2022 Nov 1. (PMC8484376; doi:10.1111/pcmr.12975)
Supplement: Table S6 [file NIHMS1723557-supplement-Table_S6.docx]

**Table S6.** Linkage analysis of the phenotype autosomal barring against markers on chromosome 1. Only female back-cross offspring with the variant allele at *MC1R* were used for analysis (*E*R(Fay)/E*N*) in order to detect contributions by different sexes. All markers with maximum LOD score> 2.0 are listed.

| **Marker** | **Position Galgal4 (bp)** | **Position Galgal6 (bp)** | **Rec.fraction** | **LOD score** |
| --- | --- | --- | --- | --- |
| M2 | 40021056 | 40174251 | 0.28 | 2.18 |
| M3 | 40571954 | 40725460 | 0.28 | 2.18 |
| M4 | 41175621 | 41328645 | 0.28 | 2.18 |
| M5 | 41769860 | 41921996 | 0.28 | 2.18 |
| M9 | 42821319 | 42972428 | 0.28 | 2.18 |
| M11 | 43533323 | 43690588 | 0.28 | 2.18 |
| M13 | 44128427 | 44286319 | 0.28 | 2.18 |
| M16 | 45076208 | 45235697 | 0.28 | 3.08 |
| M21 | 47056988 | 47237279 | 0.26 | 2.61 |
| M22 | 47368127 | 47507540 | 0.26 | 2.61 |
| M25 | 48355447 | 48498780 | 0.25 | 2.90 |
| M26 | 48718888 | 48862557 | 0.24 | 3.08 |
| M27 | 49022195 | 49165859 | 0.22 | 3.61 |
| M28 | 49136914 | 49280579 | 0.22 | 3.61 |
| M31 | 49801782 | 49944346 | 0.24 | 3.08 |
| M32 | 50117333 | 50259829 | 0.24 | 3.08 |
| M33 | 50263114 | 50405610 | 0.24 | 3.08 |
