# Supplementary material for: The feather pattern autosomal barring in chicken is strongly associated with segregation at the MC1R locus
Source: Pigment Cell Melanoma Res. Author manuscript; Available in PMC 2022 Nov 1. (PMC8484376; doi:10.1111/pcmr.12975)
Supplement: Table S9 [file NIHMS1723557-supplement-Table_S9.docx]

**Table S9.** Amino acid sequences for *MC1R/E* alleles detected by Sanger sequencing. ‘Ref’ refers to red junglefowl individual used to generate the reference genome for chicken. ‘RJF’ – red junglefowl (wild-type); ‘Fay’ – Fayoumi; ‘PRP’ – Plymouth Rock Partridge; ‘HSS’ – Hamburg Silver Spangled; ‘SS’ – Sebright Silver.

| **Population** | **Pattern** | **n** | **M71T** | **E92K** | **V126I** | **L133Q** | **H215P** | ***MC1R* genotype** |
| --- | --- | --- | --- | --- | --- | --- | --- | --- |
| **Ref** | None | 1 | M | E | V | L | H | *N*/*N* |
| **RJF** | None | 8 | - | - | - | - | - | *N*/*N* |
| **Fay** | Autosomal | 18 | - | E/K | - | L/Q | - | *R(Fay)*^/^*R* |
|  | barring | 42 | - | - | - | Q | - | *R(Fay****)***/*R(Fay)* |
| **PRP** | Pencilling | 16 | T | K | - | - | P | *B/B* |
| **HSS** | Spangling | 16 | - | K | I | - | - | *R/R* |
| **SS** | Single lacing | 16 | - | K | I | - | - | *R/R* |

n=number of individuals; -= identical to the reference sequence
