# Supplementary material for: The feather pattern autosomal barring in chicken is strongly associated with segregation at the MC1R locus
Source: Pigment Cell Melanoma Res. Author manuscript; Available in PMC 2022 Nov 1. (PMC8484376; doi:10.1111/pcmr.12975)
Supplement: Table S10 [file NIHMS1723557-supplement-Table_S10.docx]

**Table S10.** Primer sequences used in this study.

| Application | Gene | Forward primer  5->3’ | Reverse primer  5->3’ | Sequencing primer  5->3’ |
| --- | --- | --- | --- | --- |
| qPCR | ***MC1R*** | GCGCTACCACAGCATCATGA | GGTGCTGGAGACGGTGCT | None |
|  | ***NQO1*** | TGCCTCATGCTCTTCTGTTT | TAAAGGCCTCAATTTTGAGAAA | None |
|  | ***CDH1*** | ACCAGGACCAGGACTACGAC | AGGGCTGCCTATTCATCATC | None |
|  | ***WWP2*** | CCAGCCATGTGAAGATCAGT | GTCGTAGGGCTTCATGTTCA | None |
| Pyro | ***MC1R*** | CACGACGTTGTAAAACGACGCAGCTCCGTCGTGTCCT | GGTAGCGCAGCGCATAGAAG | TCCACGGCGATGACC |
|  | ***NQO1*** | CACGACGTTGTAAAACGACCCCTGCCCTTGTGCCTTAAA | GTCCCCAACAGCTGCTTC | GCCTCACAGCACCTC |
|  | ***CDH1*** | CACGACGTTGTAAAACGACCTTCCCCGTGTTGGTGCT | CTGTCCTGGGGCACACTG | TGCCTCGTCGCACCG |
